# The Role of High-Intensity Interval Training (HIIT) in Neuromuscular Adaptations: Implications for Strength and Power Development—A Review

**DOI:** 10.3390/life15040657

**Published:** 2025-04-16

**Authors:** Chi-Hsiang Hung, Chun-Hsien Su, Dong Wang

**Affiliations:** 1 Department of Ball Sports, University of Taipei, Taipei City 111036, Taiwan; rugby@utaipei.edu.tw; 2 Graduate Institute of Sport Coaching Science, Chinese Culture University, Taipei City 111396, Taiwan; 3 Sports Rehabilitation Department of Sports Teaching Department, Shanxi Medical University, Jinzhong 030604, China; 4 Graduate Institute of Sports Training, University of Taipei, Taipei City 111036, Taiwan

**Keywords:** strength development, power development, plyometric training, athletic performance

## Abstract

High-intensity Interval Training (HIIT) is increasingly recognized for enhancing neuromuscular function, strength, power, and overall athletic performance. This review systematically examined peer-reviewed studies published between 2000 and 2025, focusing on HIIT’s impact on motor unit recruitment, muscle fiber composition, neuromuscular efficiency, maximal strength, rate of force development (RFD), muscle hypertrophy, and power output. Findings indicate that HIIT significantly improves neuromuscular activation by increasing motor unit recruitment and synchronization, particularly in fast-twitch fibers essential for explosive movements. HIIT also promotes shifts toward Type II and hybrid Type IIa fibers, enhancing strength and endurance. However, despite its effectiveness in boosting RFD and explosive power, HIIT is less efficient than traditional resistance training for maximizing absolute strength and hypertrophy due to insufficient progressive overload. Integrating resistance-based and plyometric-based HIIT protocols emerged as an effective strategy to enhance strength and power concurrently. Effective periodization and recovery strategies, including active recovery and targeted nutrition, help mitigate fatigue and optimize adaptations. Notable research gaps include the long-term impacts of HIIT on neuromuscular function and the efficacy of individualized HIIT protocols based on specific athlete characteristics. Future research should focus on refining HIIT protocols for different sports, exploring its synergy with traditional resistance training, and assessing long-term adaptations to sustain strength and power gains. HIIT presents a valuable, time-efficient complement to conventional training methods for improving strength, power, and neuromuscular efficiency.

## 1. Introduction

### 1.1. Background on Neuromuscular Adaptations and Their Importance

Neuromuscular adaptations refer to the physiological and structural changes resulting from training that enhance the interaction between the nervous system and muscles, improving force production, movement efficiency, and athletic performance. These adaptations include enhanced motor unit recruitment, synchronization, firing rate, muscle fiber composition, and neuromuscular coordination, which are crucial for strength and power development [1,2].

High-load resistance training (e.g., 80% 1 RM) induces superior neural adaptations compared to low-load training (e.g., 30% 1 RM), as evidenced by increased voluntary activation and electromyographic (EMG) amplitude during maximal force production. These findings suggest that high-load training more effectively improves the nervous system’s ability to recruit and activate motor units, leading to more significant strength gains despite similar muscle hypertrophy levels between high- and low-load training [1,2]. Additionally, electrostimulation resistance training can shift muscle fiber composition towards Type I fibers, benefiting endurance performance [3]. Neuromuscular adaptations also enhance coordination and efficiency in muscle recruitment patterns, which is particularly significant for repetitive and explosive movements in sports like running and cycling. Highly trained athletes exhibit more refined muscle recruitment patterns than novices, contributing to improved movement efficiency and performance output [4,5].

These adaptations are vital for sports requiring rapid acceleration, maximal force output, and sustained high-intensity efforts, such as rugby, football, basketball, weightlifting, and sprinting, where strength and power are key performance factors. Strength is essential for lifting, pushing, tackling, and jumping, while power is critical for sprinting, striking, and explosive directional changes. High levels of both attributes directly enhance sprint speed, vertical jump height, and tackling efficiency [6,7,8]. Moreover, improved neuromuscular control and muscle stability contribute to injury prevention by reducing the risk of musculoskeletal injuries [7,8,9]. By adopting training methods that optimize neuromuscular adaptations, athletes can significantly enhance strength, power, and overall performance, making these adaptations a fundamental aspect of sports science and high-performance training [9,10,11].

### 1.2. Overview of High-Intensity Interval Training (HIIT)

High-intensity interval Training (HIIT) is a structured exercise method involving short bursts of high-intensity effort (typically 20 s to a few minutes at 80–95% of maximal heart rate), alternated with low-intensity recovery periods [12]. This review focuses specifically on HIIT and its primary protocol variations—sprint-based, resistance-based, and plyometric-integrated formats—while other training methods are only discussed for comparative context. Designed to maximize cardiovascular, metabolic, and neuromuscular adaptations more efficiently than traditional endurance training, HIIT enhances both anaerobic and aerobic energy systems, leading to improved muscle strength, power, and endurance [13,14,15,16]. Key principles of HIIT include intensity management and work-to-rest ratios, where high-intensity phases demand near-maximal effort, and recovery intervals are adjusted based on specific training goals. HIIT is highly adaptable, incorporating sprinting, cycling, bodyweight drills, resistance exercises, and plyometrics to suit various athletic needs [12]. Neuromuscular adaptations from HIIT involve enhanced motor unit recruitment, Type II muscle fiber transformations, and improved energy system efficiency, contributing to greater strength, power, and endurance [17,18,19].

Unlike traditional strength training, which focuses on progressive overload and maximal force output with more extended rest periods, HIIT simultaneously integrates high-intensity dynamic movements that challenge the cardiovascular and neuromuscular systems [20]. While strength training develops maximal force production, HIIT emphasizes shorter recovery to maintain elevated heart rates and metabolic demands, enhancing neuromuscular efficiency, power endurance, and energy utilization [21]. Compared to traditional endurance training, which primarily targets the aerobic system through prolonged, moderate-intensity efforts (60–70% HRmax), HIIT engages both anaerobic and aerobic pathways, resulting in faster cardiovascular adaptations, improved muscle oxygen utilization, and higher power output. Studies indicate that HIIT can match or surpass traditional endurance training in enhancing endurance, strength, and power within a shorter timeframe [16,21,22].

Integrating HIIT with conventional strength and endurance training helps athletes build a well-rounded physical profile, optimizing strength, power, agility, and metabolic efficiency. This approach enhances sports performance and aids in injury prevention by improving muscle stability and neuromuscular control, making HIIT a valuable component of high-performance training programs [22].

### 1.3. Purpose of the Review

This review examines the role of HIIT in neuromuscular adaptations, focusing on motor unit recruitment, muscle fiber composition, and neuromuscular efficiency in strength and power development. HIIT enhances motor unit activation, Type II fiber recruitment, and neuromuscular coordination, all of which contribute to more excellent force production, explosive power, and movement efficiency. Its applications extend beyond athletic performance to rehabilitation, aiding muscle activation, movement control, and injury recovery. However, despite the growing body of literature on HIIT and neuromuscular adaptations, few reviews systematically address the gaps in the literature regarding the use of HIIT in specialized populations such as youth athletes and patients undergoing rehabilitation. This review aims not only to synthesize current knowledge but also to highlight underexplored areas for future research.

## 2. Literature Selection and Scope

This review adopts a narrative approach to synthesize recent evidence on the neuromuscular adaptations induced by High-Intensity Interval Training (HIIT), specifically focusing on strength and power development. The objective is to provide an integrative overview of current findings, highlight key mechanisms, and discuss practical applications for athletes and practitioners.

Relevant literature was identified through a targeted search of peer-reviewed journal articles published between 2000 and 2025, using databases such as PubMed, Scopus, Web of Science, and EBSCO. Search terms included combinations of the following keywords: “high-intensity interval training”, “HIIT”, “neuromuscular adaptations”, “motor unit recruitment”, “strength development”, “power output”, and “athletic performance”.

Articles were selected based on their relevance to the review’s core themes: (1) the physiological and neuromuscular responses to HIIT; (2) strength and power outcomes in athletic populations; and (3) training methods involving sprint-based, resistance-based, and plyometric-based HIIT protocols.

Both original research articles and review papers were considered to ensure a broad representation of current knowledge. Studies involving both trained and untrained athletes were included. The selection emphasized findings applicable to sports performance enhancement, rather than clinical rehabilitation or general fitness.

Rather than following a systematic inclusion/exclusion framework, this review emphasizes conceptual integration and thematic synthesis, drawing connections between neuromuscular principles and HIIT methodologies. Key themes were organized into sections on motor unit recruitment, muscle fiber adaptations, neuromuscular efficiency, strength and power development, and training strategies.

This approach allows for a more flexible and comprehensive discussion of emerging trends, gaps in the literature, and practical implications for strength and conditioning.

## 3. Mechanisms of Neuromuscular Adaptations to HIIT

High-intensity interval training promotes neuromuscular adaptations that enhance motor unit recruitment, muscle fiber composition, neuromuscular efficiency, and metabolic function, improving strength, power, and endurance. These adaptations, driven by neural, muscular, and metabolic mechanisms, collectively boost force production, movement efficiency, and fatigue resistance, offering valuable insights into how HIIT optimizes performance for athletes across various sports. Unless otherwise specified, the following mechanisms are synthesized from the 86 studies included in our systematic search.

### 3.1. Motor Unit Recruitment and Neural Drive

Effective at inducing neuromuscular adaptations, HIIT enhances motor unit recruitment, synchronization, and firing frequency. Research demonstrates that HIIT significantly increases the activation of fast-twitch fibers, which are essential for explosive movements such as sprinting and jumping. The intense demands of HIIT improve recruitment efficiency by engaging a more significant number of motor units [23,24]. Additionally, HIIT promotes better synchronization of motor units, leading to more coordinated muscle contractions, which is critical for rapid and robust movements [25]. By repeatedly targeting a broad pool of fast-twitch fibers, HIIT enhances explosive power enabling muscles to generate force quickly—an essential factor for short-burst activities that rely on RFD [24,26]. The impact on neural drive is another significant benefit, as HIIT improves signal transmission efficiency from the central nervous system (CNS) to the muscles. This enhancement leads to faster and more synchronized muscle contractions, reducing reaction times and improving performance efficiency [27,28]. These improvements are linked to increased motor unit discharge rates and lower recruitment thresholds, facilitating more effective muscle activation during high-intensity efforts [29]. Both studies and meta-analyses confirm that HIIT significantly boosts sprint speed, aerobic capacity, and overall neuromuscular function, supporting its value as a training strategy for athletes seeking to enhance strength, power, and performance [21,23]. These findings underscore HIIT’s effectiveness in optimizing neuromuscular adaptations, making it a key component of high-performance training.

### 3.2. Muscle Fiber Type Adaptations

Significant neuromuscular adaptations from HIIT include an increase in Type II (fast-twitch) fibers and a shift from Type I (slow-twitch) fibers, enhancing explosive power and high-intensity performance—key for sports like basketball, soccer, and rugby [30,31]. This adaptation contrasts with traditional endurance training, primarily developing Type I fibers for aerobic performance. A notable advantage of HIIT is the development of hybrid fibers (Type IIa), which combine the strength of fast-twitch fibers with the fatigue resistance of slow-twitch fibers, enabling athletes to sustain high power outputs over longer durations [31]. This shift enhances force output, sprinting ability, and muscular endurance, allowing athletes to perform repeated high-intensity efforts effectively [31]. However, a balanced training approach is crucial. Traditional endurance training remains essential for sports requiring sustained aerobic capacity while integrating resistance training with HIIT can further optimize muscle adaptations. Studies on combined training protocols suggest superior improvements in both strength and endurance [32]. This evidence supports a comprehensive training strategy incorporating HIIT, resistance training, and endurance exercises to maximize explosive power and sustained performance, ensuring a well-rounded athletic profile.

### 3.3. Neuromuscular Efficiency and Coordination

Enhancing neuromuscular efficiency is a key benefit of HIIT, achieved through improved intramuscular and intermuscular coordination. This leads to synchronized muscle fiber activity and more efficient force transfer across joints, optimizing force production with minimal energy expenditure [23,33]. HIIT improves muscle fiber synchronization, resulting in smoother and more powerful contractions essential for explosive movements like sprinting and jumping. Studies confirm that HIIT significantly enhances muscle strength and explosive power, which are critical for sports performance [23,33]. Additionally, HIIT refines the coordination between multiple muscle groups during complex movements, enhancing force transfer in multi-joint actions such as weightlifting [23,30]. By progressively exposing the nervous system to high-intensity loads, HIIT trains the body to override inhibitory responses, enabling more excellent force production and movement execution under fatigue—crucial for competitive scenarios [16,21]. Reducing neuromuscular inhibition through HIIT also supports higher levels of force output, as evidenced by improvements in sprint performance and maximal aerobic power [16,24]. These findings highlight HIIT’s effectiveness in enhancing neuromuscular efficiency, coordination, and power, making it a valuable training method for athletes seeking to maximize performance across various sports.

### 3.4. Metabolic and Structural Adaptations

High-Intensity Interval Training (HIIT) induces significant metabolic and structural adaptations that enhance energy production and endurance. Key adaptations include increased mitochondrial density, improved oxidative enzyme activity, and enhanced capillarization, collectively improving ATP generation, oxygen utilization, and nutrient transport to muscles [34]. Greater mitochondrial density enables muscles to sustain repeated bursts of power and delay fatigue, making HIIT effective for high-intensity activities [35]. HIIT also improved mitochondrial function and prevented metabolic dysfunctions by enhancing beta-oxidation and insulin sensitivity [36]. Additionally, enhanced oxidative enzyme activity improves the muscle’s ability to utilize oxygen efficiently, facilitating faster recovery between efforts [34]. Research combining HIIT with blood-flow restriction showed significant gains in maximal aerobic power and sprint performance, underscoring the role of oxidative enzymes in optimizing aerobic capacity [16]. Furthermore, HIIT promotes capillarization, improving blood flow, oxygen delivery, and nutrient transport to muscles, supporting aerobic and anaerobic energy systems [12]. Increased capillary density helps maintain high-performance levels and accelerates recovery [34]. These adaptations are crucial for sustaining high-intensity efforts and enhancing recovery between sessions, making HIIT an effective strategy for improving endurance and overall performance.

In addition to the acute and mid-term neuromuscular adaptations discussed earlier, several studies have examined the long-term effects of HIIT on sustained improvements in strength and power. These long-term adaptations appear to be influenced by individual characteristics, including genetic predispositions, muscle fiber composition, and recovery capacity, highlighting the importance of personalized programming approaches [32]. Moreover, when adequately periodized, the integration of HIIT with traditional strength and plyometric training has been shown to enhance both power output and endurance capacity without causing interference effects. This supports the role of HIIT as a valuable complementary component within comprehensive performance training frameworks [26]. In rehabilitation contexts, HIIT has also demonstrated the potential to restore neuromuscular function following injury or periods of immobilization. Evidence suggests that structured HIIT protocols can enhance motor unit recruitment, proprioceptive control, and muscle reactivation, making it a practical option for early-phase return-to-play strategies [10,11]. Additionally, the time-efficient nature of HIIT may promote better psychological engagement and support progressive recovery in post-injury populations.

HIIT induces neuromuscular adaptations that significantly enhance strength, power, and endurance, making it highly effective for power-based and intermittent sports. HIIT improves explosive strength, movement efficiency, and fatigue resistance by optimizing motor unit recruitment, muscle fiber composition, neuromuscular coordination, and metabolic function. These adaptations elevate athletic performance and contribute to injury prevention and muscular resilience, highlighting HIIT’s versatility in sports training and rehabilitation. A detailed description of these neuromuscular adaptations to HIIT is provided in Table 1.

## 4. HIIT and Strength Development

Increasing recognition surrounds HIIT for its ability to enhance neuromuscular performance in strength and power-based sports. Though primarily associated with aerobic and anaerobic conditioning, HIIT can support strength development when adequately structured. However, its effects on maximal strength, rate of RFD, and hypertrophy differ from traditional resistance training, emphasizing the need to carefully evaluate its advantages, limitations, and integration strategies.

### 4.1. Effects of HIIT on Maximal Strength

Maximal strength—the maximum force a muscle or group can generate—is crucial for athletic performance, injury prevention, and physical resilience [9,22]. HIIT supports strength development by promoting high-intensity contractions that activate motor units and fast-twitch fibers responsible for force production [37]. Sprint-based HIIT (SIT) effectively engages the posterior chain muscles (glutes, hamstrings, lower back), enhancing lower body strength and explosive power essential for activities requiring rapid force production [37]. Resistance-based HIIT, incorporating exercises like kettlebell swings, jump squats, and sled pushes, targets multiple muscle groups, boosting overall strength and neuromuscular coordination [38]. However, unlike traditional resistance training, HIIT generally lacks progressive overload with heavy external loads, limiting its ability to maximize absolute strength and hypertrophy. The systematic load increases in conventional resistance training drive more substantial neuromuscular adaptations, making it superior for achieving greater muscle strength [39]. Maximal strength is strongly linked to performance in dynamic activities (e.g., countermovement jumps), where traditional resistance training excels due to its emphasis on heavy lifting and a full range of motion [40]. Thus, while HIIT can significantly improve strength and power, a balanced approach integrating HIIT and traditional resistance training is likely the most effective strategy for optimizing athletic performance.

### 4.2. HIIT’s Impact on Rate of Force Development (RFD)

The RFD, the speed at which force is produced, is vital for explosive movements such as sprinting, jumping, and tackling. HIIT significantly enhances neuromuscular factors like the H-reflex and V-wave, which are crucial for explosive strength and correlate positively with RFD, indicating improved neural drive and muscle activation efficiency [41,42]. Corticospinal excitability, a key determinant of RFD, is also enhanced through HIIT, as shown by increased motor-evoked potentials during explosive contractions [43]. HIIT enhances vertical stiffness—the ability to generate and transmit force rapidly—through depth jumps and ballistic movements, leading to better force transmission and rapid force generation [42]. Tendon stiffness, especially in the Achilles tendon, is improved by HIIT, enhancing elastic energy storage and release, which supports explosive movements [41]. Resisted sprints and plyometric exercises within HIIT protocols effectively stimulate fast-twitch muscle fibers, which is essential for rapid force production and improved explosive performance [44,45]. The short recovery intervals in HIIT simulate game-like conditions, training athletes to maintain force production under fatigue, a critical factor for sustaining RFD [46]. HIIT should be periodized with maximal strength training for optimal long-term RFD improvements, as increasing absolute strength provides a foundation for explosive power [45]. Thus, HIIT’s ability to enhance neuromuscular efficiency, tendon properties, and fast-twitch fiber activation makes it an effective strategy for improving RFD and explosive athletic performance.

### 4.3. Potential Limitations of HIIT for Strength Gains

HIIT, with its high metabolic demand and short recovery periods, can hinder muscle recovery and force production, limiting hypertrophy and maximal strength gains. In contrast, traditional strength training allows longer rest intervals, promoting more effective recovery and sustained force production [12,34]. HIIT’s emphasis on speed and endurance may also reduce mechanical tension and high-load fiber recruitment, which is essential for maximal strength development [47]. However, combining HIIT with resistance training (RT) yields superior fitness outcomes. Studies show concurrent HIIT and RT enhance cardiorespiratory fitness and muscle mass more effectively than HIIT alone [32,38]. While HIIT cannot replace maximal strength training, it is an effective complement during the power, speed, and endurance phases. Its ability to improve neuromuscular efficiency and cardiovascular fitness makes it a valuable part of a comprehensive training program [48,49]. Strategic integration of HIIT can optimize strength and endurance without compromising maximal force production.

While HIIT may be less effective for developing absolute maximal strength, it substantially improves the rate of force development (RFD), neuromuscular efficiency, and explosive force production—key factors for athletic performance. HIIT is a valuable supplementary method for enhancing dynamic strength, power, and movement efficiency in high-intensity sports when integrated with traditional strength training. A comprehensive overview of HIIT’s impact on strength development is presented in Table 2.

## 5. HIIT and Power Development

Power, defined as the ability to generate maximum force rapidly, is essential for explosive movements like sprinting, jumping, and tackling. When properly structured, HIIT effectively enhances power output by improving neuromuscular activation, RFD, and reactive strength through sprint-based and plyometric protocols. Understanding HIIT-induced power adaptations compared to traditional power training provides valuable insights for optimizing explosive performance. Unless otherwise stated, the outcomes discussed in this section reflect the evidence synthesized from the 86 systematically reviewed studies.

### 5.1. Enhancing Explosive Power Through HIIT

Explosive power, determined by motor unit recruitment, muscle fiber composition, and neuromuscular efficiency, is significantly enhanced by HIIT through short, high-intensity efforts that improve force production speed. Sprint-based HIIT, including all-out sprints, resisted sprints, and sled pushes, effectively boosts horizontal force application, which is critical for acceleration and sprint speed [26,41]. Studies confirm that sprint training enhances the rate of force development (RFD) and neuromuscular efficiency, optimizing sprint performance [26,41]. Numerical simulations suggest that the quadriceps and gastrocnemius muscles play a vital role in horizontal propulsion during sprints [50]. HIIT also increases α-motoneuron excitability and muscle strength, which is essential for explosive movements [41]. Additionally, plyometric exercises like box jumps and depth jumps improve vertical force application and reactive strength, enhancing vertical stiffness and peak force during countermovement jumps, which are linked to better explosive performance [33,42]. Studies show that plyometric training improves vertical jump height and sprint times, enhancing explosive power and neuromuscular coordination [33,51]. Combining high-intensity efforts with minimal recovery in HIIT also boosts fatigue resistance, allowing athletes to sustain power output over multiple efforts. Integrating sprint-based HIIT and plyometrics into training effectively enhances explosive power, neuromuscular efficiency, and fatigue resistance, making it a powerful strategy for athletic performance improvement.

### 5.2. Plyometric-Based HIIT and Power Gains

Combining plyometric training with HIIT effectively enhances explosive power and athletic performance by leveraging the stretch-shortening cycle (SSC) to improve neuromuscular activation, tendon stiffness, and intermuscular coordination—key for sports requiring rapid force generation [33,52]. Plyometric exercises like jump squats and box jumps activate fast-twitch fibers through rapid eccentric-concentric actions, promoting explosive movements [33,52].

Improved tendon stiffness from plyometric training enhances elastic energy storage and release, enabling faster, more powerful movements [53,54]. Additionally, intermuscular coordination is refined, aiding in quick direction and speed changes [55,56]. Studies confirm that plyometric training significantly increases vertical jump height, sprint acceleration, and change-of-direction speed, mainly due to enhanced SSC efficiency [52,55]. Combining plyometric training with resistance training (RT) further optimizes performance metrics, suggesting a mixed training approach offers the most significant benefits for enhancing explosive strength and agility [33,57]. This integrated strategy supports balanced improvements in power and endurance, making it ideal for high-performance sports training.

### 5.3. Comparison with Traditional Power Training Methods

HIIT and Olympic weightlifting effectively enhance athletic performance but differ in their approach and outcomes. Olympic weightlifting requires high technical skill and more extended recovery periods, making it ideal for developing maximal power and strength, particularly in the lower body, which benefits jumping and sprinting [58,59]. In contrast, HIIT, especially with plyometric exercises, is more accessible and can be tailored to sport-specific movements, enhancing anaerobic capacity and neuromuscular efficiency [26,60]. A strategic approach involves focusing on Olympic lifts to build power in the off-season, followed by integrating HIIT-based plyometrics to enhance power endurance and sport-specific explosiveness [21,60]. Alternating heavy resistance and explosive HIIT drills can also amplify post-activation potentiation (PAP), boosting force output and acceleration [59]. Research shows that HIIT significantly improves speed and aerobic and anaerobic capacity, making it essential in a comprehensive training program [26,61]. Combining HIIT with low-intensity training in structured HIIT shock microcycles further enhances endurance and recovery [62]. Thus, an integrated approach that combines the strength benefits of Olympic weightlifting with the conditioning advantages of HIIT offers well-rounded improvements in athletic performance.

HIIT significantly improves power development by enhancing fast-twitch fiber activation, neuromuscular coordination, and fatigue resistance through sprint-based and plyometric training. Although traditional power training methods, such as Olympic lifting, are essential for maximizing absolute power, HIIT is an effective complement by facilitating explosive training under high-intensity conditions. Strategic integration of HIIT within strength-power cycles optimizes force production, sustained power output, and overall sports performance across various disciplines. A detailed overview of HIIT’s impact on power development is presented in Table 3.

## 6. Training Implementation: HIIT Protocols for Strength and Power Gains

Effective HIIT for strength and power development requires strategic protocol selection to optimize neuromuscular adaptations, explosive force production, and movement efficiency. The structure of HIIT—including work duration, rest intervals, and session frequency—is a fundamental determinant of the resulting neuromuscular and physiological adaptations. For instance, sprint-based HIIT typically employs 6–10 s of maximal effort followed by 30–60 s of rest, primarily targeting ATP-PCr recovery. Resistance-based HIIT often uses 20–40 s of effort with shorter rest to emphasize muscular endurance and fatigue resistance. These timing parameters condition the adaptive responses, such as increased tendon stiffness, hypertrophy, or explosive force output, and must match the athlete’s goals and training phase. Unlike endurance-focused HIIT, power-based HIIT should emphasize maximal effort, short-duration intervals, and adequate recovery to prevent excessive fatigue. Sprint Interval Training (SIT), resistance-based HIIT, and plyometric-integrated HIIT effectively develop power, strength, and sport-specific explosiveness. Protocols must align with athlete needs, sports demands, and periodization to complement rather than hinder traditional strength training. The training strategies and protocols summarized in this section are drawn from the studies identified in the systematic search.

### 6.1. Sprint Interval Training (SIT) for Power Development

Sprint Interval Training (SIT) effectively enhances power, speed, and anaerobic capacity by improving neuromuscular performance, critical for acceleration and explosive force [63]. Studies show that SIT significantly improves linear speed and change in direction ability, particularly in novice sprinters, enhancing initial sprint performance [63,64]. SIT also boosts anaerobic capacity, evidenced by increased peak and average power outputs in the Wingate test for tennis players [65]. A progressive sprint-release model of HIIT, incorporating SIT, has been shown to significantly enhance anaerobic capacity in rugby athletes, highlighting its role in optimizing energy system efficiency [26]. Effective SIT protocols typically involve 6–10 s of maximal sprints followed by 30–60 s of rest, optimizing ATP-PCr recovery and maintaining high force production [66]. Although SIT improves muscle glycolytic content and activity, it may not significantly extend endurance during severe-intensity exercise [67]. This suggests that SIT is more effective for short-burst power and anaerobic performance than for enhancing prolonged endurance.

### 6.2. Resistance-Based HIIT Protocols

Resistance-based HIIT effectively enhances strength and power adaptations through explosive, weighted movements. Integrating resistance training into HIIT improves lower body explosive strength and maximal strength, as shown by enhanced countermovement jump (CMJ) and half squat performance, with longer durations favoring CMJ and shorter durations benefiting half squat [38]. High-intensity power training (HIPT), a variant of resistance-based HIIT, has been shown to improve upper and lower limb explosive force more efficiently than traditional resistance training while enhancing mean anaerobic power [68]. Olympic lifting, a key element of resistance-based HIIT, significantly develops power, benefiting speed, strength, and overall performance in sports like football [69]. Contrast training, which alternates heavy resistance with explosive movements, leverages PAP to improve neuromuscular efficiency and force production during high-speed movements [70]. Additionally, kettlebell exercises enhance power qualities and cardiovascular function while inducing a significant acute hormonal response that supports muscle adaptations and fitness [71,72].

### 6.3. Plyometric and Agility-Integrated HIIT

Plyometric-based HIIT effectively enhances elastic strength, reactive power, and rapid force production, which is essential for sports involving stretch-shortening cycles (SSC). When combined with sprint exercises, plyometric training significantly improves sprint speed and change-of-direction speed, as demonstrated by enhanced agility and performance in youth soccer players [73]. This training also boosts jumping abilities—vertical, lateral, and horizontal—crucial for sports like basketball by enhancing the rate of force development and ground reaction force [52,57]. Plyometrics increase explosive strength and speed performance for track and field athletes, emphasizing its effectiveness [55]. Additionally, plyometric training enhances reactive strength and proprioceptive control, which are vital for rapid and precise movements [45]. Long-term high-intensity plyometrics significantly boost lower-body strength and power, supporting performance across various sports [74]. Furthermore, it aids injury prevention by strengthening lower-limb muscles and enhancing joint stability, as seen in reduced injury rates among basketball players [51]. Effective implementation and monitoring, considering individual athlete needs, are crucial for optimizing results and ensuring safety.

### 6.4. Sport-Specific HIIT Applications

HIIT is an adaptable training method that effectively enhances speed, aerobic, and anaerobic capacity to meet the specific demands of various sports. Exercises like sled pushes and grappling drills replicate the demands of contact sports, improving power and sport-specific strength [21]. Short-duration, high-intensity efforts are essential for sports like rugby and American football, which require explosive power bursts [26]. Multi-directional sprints enhance agility and speed, which is critical for basketball and soccer [21]. Incorporating sport-specific drills helps athletes sustain performance under fatigue, ensuring a competitive advantage [26]. For sports like weightlifting and sprinting, HIIT should emphasize short, intense bursts with adequate recovery to preserve power output and prevent fatigue accumulation [21,26].

Implementing HIIT effectively for strength and power gains requires careful selection of exercises, appropriate work-to-rest ratios, and sport-specific modifications. Sprint-based, resistance-based, and plyometric-integrated HIIT protocols enhance neuromuscular activation, explosive force, and fatigue resistance, making them highly beneficial for athletic training. However, to maximize power development, HIIT should complement traditional strength training and be integrated into a well-structured periodization plan. Evidence-based HIIT protocols allow athletes to develop balanced strength, power, and endurance, improving sport-specific performance and resilience. A comprehensive summary of HIIT protocols for strength and power gains is presented in Table 4.

## 7. Practical Considerations and Periodization Strategies

Strategic planning, periodization, and recovery are crucial for optimizing HIIT’s impact on strength and power development. While HIIT effectively enhances neuromuscular efficiency, power, and endurance, improper integration with strength training can cause fatigue accumulation and interference, impeding long-term gains. Balancing HIIT with traditional strength training and tailoring protocols to individual needs are essential for maximizing performance adaptations.

### 7.1. Balancing HIIT with Strength Training

Integrating HIIT with strength training requires strategic planning to minimize interference effects and optimize strength and power gains. Short-duration, high-intensity efforts in HIIT can enhance neuromuscular activation, particularly when combined with blood-flow restriction, which has been shown to improve sprint performance and anaerobic adaptations without significantly affecting cardiorespiratory fitness [16]. HIIT should moderate volume and intensity to prevent excessive fatigue, especially during competition phases [75]. Scheduling HIIT separately from or after strength training maximizes force production during resistance exercises, minimizing fatigue-related impairments [75]. Additionally, managing lower-body intensive HIIT is essential to prevent overloading muscle groups involved in resistance training, with adequate recovery being crucial to avoiding interference effects [76].

### 7.2. Fatigue Management and Recovery Strategies

HIIT is a potent stimulus for physiological adaptation but also imposes significant neuromuscular fatigue. Heart Rate Variability (HRV) and Rate of Perceived Exertion (RPE) are adequate for assessing recovery status, with HRV providing insights into autonomic recovery and RPE indicating perceived exertion levels [77]. Neuromuscular fatigue can be evaluated through CMJ and grip strength tests, with CMJ performance typically declining post-exercise and recovering within 48–72 h [78]. Active recovery methods, such as alternating hot and cold water immersion, enhance blood flow, reduce muscle soreness, and expedite recovery [77]. Nutritional strategies, including adequate protein and carbohydrate intake, support muscle repair and glycogen replenishment, while hydration and quality sleep are critical for hormonal recovery and neuromuscular efficiency [79]. HIIT induces central and peripheral fatigue, impacting neural function and muscle contractility. Yet, when managed correctly, it significantly enhances aerobic capacity and performance metrics such as VO2 max and repeated-sprint ability [21,25,80,81].

### 7.3. Individualization of HIIT Protocols

Effectively optimizing HIIT for strength and power development depends on tailoring protocols to the individual athlete’s profile, including their training history, current physical condition, and specific performance goals. Lower-intensity HIIT with longer recovery suits beginners, while elite athletes benefit from high-intensity protocols with shorter recoveries that simulate competition [45]. Sport-specific adaptations are also essential; resistance-based HIIT can enhance strength under fatigue for contact sports, while sprint and plyometric HIIT effectively improve RFD and acceleration for speed-based sports [45,82]. Adjusting work-to-rest ratios based on sport-specific demands is essential. For example, high work-to-rest ratios (1:3 or greater) favor recovery and power output in sprint protocols, while lower ratios (1:1 or 2:1) can enhance metabolic conditioning and fatigue resistance. Individual responses to these ratios should guide HIIT design, particularly for optimizing strength, hypertrophy, or reactive power. Individualizing HIIT using metrics like anaerobic speed reserve (ASR) and anaerobic power reserve (APR) has been shown to promote uniform physiological adaptations among athletes with diverse profiles [83,84]. Integrating HIIT with strength training can further enhance aerobic fitness and match performance, underscoring the importance of a comprehensive approach [85]. Periodized HIIT, adjusted for intensity, volume, and recovery, maximizes neuromuscular adaptations and minimizes injury risk, supporting sustained performance improvements [26,86].

Strategic HIIT periodization effectively maximizes neuromuscular adaptations while preserving strength and power gains. Combining HIIT with strength training, implementing effective fatigue management, and customizing protocols based on individual needs can significantly enhance explosive performance, endurance, and injury resilience. When applied thoughtfully, HIIT is a complementary approach to strength training, creating a synergistic effect that optimizes long-term performance and power output. A detailed overview of the key focus on HIIT practical considerations and periodization strategies is presented in Table 5.

## 8. Future Research Directions

The long-term effects of HIIT on neuromuscular function, strength, and power remain insufficiently understood, as most studies emphasize short-term adaptations. Examining sustained improvements in maximal strength, power output, and muscle hypertrophy through extended HIIT protocols, in comparison to traditional resistance training, is crucial. Equally important is the investigation of potential risks associated with long-term HIIT application, including injury susceptibility, neuromuscular fatigue accumulation, and overtraining, especially when protocols are not adequately individualized or periodized. Additionally, assessing risks such as overuse injuries, neuromuscular fatigue, and adaptive plateaus could refine periodization strategies, ensuring that HIIT is implemented safely and effectively for power-dominant sports. Understanding individual variability in response to HIIT—shaped by genetics, muscle fiber composition, and training history—may allow for more personalized protocols that enhance power adaptations while minimizing fatigue and interference effects. In addition, the application of HIIT in children and adolescent athletes remains an understudied area. Considering the developmental differences in neuromuscular systems and recovery profiles, future research should investigate age-appropriate HIIT protocols, neuromuscular outcomes, and safety implications in pediatric populations.

Exploring optimal HIIT protocols for power-based sports like weightlifting, sprinting, and combat sports is also essential. Future research should also stratify findings based on HIIT methodology, including variations in work duration, rest intervals, and intensity levels. Differentiating these variables will allow a more precise understanding of how specific HIIT formats drive distinct neuromuscular adaptations. Evaluating the efficacy of sprint interval training (SIT), plyometric-integrated HIIT, and resistance-based HIIT could provide more precise guidelines for maximizing power output. Integrating HIIT with traditional strength, hypertrophy, and plyometric training requires careful consideration to avoid interference effects. With appropriate adjustments to HIIT intensities, frequencies, and work-to-rest ratios, strategic periodization may help maintain strength gains while enhancing power. Future research should also explore monitoring tools and recovery strategies that support the safe and sustainable implementation of HIIT over the long term. Advancing these research areas can provide valuable insights for coaches and athletes, ensuring that HIIT is a powerful tool for sustainable improvements in strength, power, and overall performance.

## 9. Conclusions

High-Intensity Interval Training (HIIT) has proven to be a versatile and effective method for enhancing neuromuscular function, strength, power, and endurance. Its ability to optimize motor unit recruitment, muscle fiber composition, and neuromuscular efficiency underscores its value for power-based and intermittent sports. Maximizing the benefits of HIIT while minimizing potential interference effects with traditional resistance training requires careful planning, periodization, and individualized programming. Strategic integration of HIIT with strength, hypertrophy, and plyometric training can result in comprehensive improvements in explosive force production, fatigue resistance, and overall athletic performance. Continued research into HIIT’s long-term effects, individualized responses, and optimal protocols will further refine its application in high-performance training programs, enabling athletes to achieve sustainable gains in strength, power, and sport-specific abilities.

## Figures and Tables

**Table 1:** Neuromuscular adaptations to HIIT.

Aspect	Key Adaptations	Detailed Findings	References
Motor Unit Recruitment and Neural Drive	Enhanced recruitment, synchronization, and firing frequency of motor units. Improved neural drive.	HIIT significantly increases the activation of fast-twitch fibers, improving the explosive power and rate of RFD. Enhances motor unit synchronization for coordinated contractions. Boosts neural drive efficiency, reducing reaction time.	[23,24,25,26,27,28,29]
Muscle Fiber Type Adaptations	Increased Type II fibers and hybrid Type IIa fibers. Shift from Type I to Type II fibers.	HIIT promotes a shift to fast-twitch fibers, enhancing explosive power. Hybrid fibers (Type IIa) combine strength and endurance properties, enabling sustained high-power outputs.	[30,31,32]
Neuromuscular Efficiency and Coordination	Improved intramuscular and intermuscular coordination. Enhanced force transfer efficiency.	HIIT refines muscle synchronization, improving force production and movement efficiency. Reduces neuromuscular inhibition, supporting greater force output.	[16,21,23,30,33]
Metabolic and Structural Adaptations	Increased mitochondrial density, oxidative enzyme activity, and capillarization.	HIIT enhances ATP production, oxygen utilization, and nutrient transport. It improves mitochondrial function and prevents metabolic dysfunctions. Promotes capillarization for better blood flow and recovery.	[12,16,34,35,36]

Abbreviations: RFD = rate of force development; HIIT = high-intensity interval training.

**Table 2:** HIIT and strength development.

Aspect	Key Findings	Detailed Insights	References
Effects of HIIT on Maximal Strength	HIIT supports strength development through high-intensity contractions activating fast-twitch fibers.	Sprint-based HIIT engages posterior chain muscles, enhancing lower body strength and explosive power. Resistance-based HIIT improves strength and coordination using kettlebell swings, jump squats, and sled pushes. Lacks progressive overload, limiting maximal strength and hypertrophy compared to traditional resistance training.	[37,38,39,40]
HIIT’s Impact on RFD	Enhances neuromuscular factors like H-reflex and V-wave, improving RFD.	HIIT increases corticospinal excitability and motor-evoked potentials, enhancing neural drive. Improves tendon stiffness, elastic energy storage, and release, supporting explosive movements. Short recovery intervals train force production under fatigue, simulating game-like conditions.	[41,42,43,44,45,46]
Potential Limitations of HIIT for Strength Gains	HIIT may hinder recovery and maximal strength due to short recovery periods.	Emphasis on speed and endurance reduces mechanical tension and high-load fiber recruitment. Combining HIIT with resistance training enhances cardiorespiratory fitness and muscle mass. It is effective as a complement to traditional strength training, not a replacement.	[12,32,34,37,47,48,49]
Overall Impact of HIIT on Strength and Power	HIIT enhances RFD, neuromuscular efficiency, and explosive power.	Optimizes motor unit recruitment, muscle fiber composition, and movement efficiency. Valuable for dynamic strength and power when integrated with traditional strength training.	[37,38,39,41,42,45]

**Table 3:** HIIT and power development.

Key Focus	Key Findings	Training Methods	Benefits	Limitations	References
Enhancing Explosive Power Through HIIT	HIIT significantly enhances explosive power by optimizing motor unit recruitment, muscle fiber composition, and neuromuscular efficiency. Sprint-based HIIT, including sprints, resisted sprints, and sled pushes, improves horizontal force application and acceleration speed. Plyometric exercises enhance vertical force and reactive strength, leading to better vertical stiffness and peak force.	Sprint-based HIIT, Plyometric HIIT	Improves RFD, power output, and sprint performance	Limited impact on maximal strength due to lack of heavy progressive overload	[26,33,41,42,51]
Plyometric-Based HIIT and Power Gains	Combining plyometric training with HIIT leverages the stretch-shortening cycle (SSC) to improve neuromuscular activation, tendon stiffness, and intermuscular coordination. Plyometric exercises target fast-twitch fibers through rapid eccentric-concentric actions. Improved tendon stiffness aids in faster and more powerful movements.	Plyometric HIIT, Resistance-based HIIT	Enhances SSC efficiency, jump height, and direction-change speed	Requires precise programming to avoid fatigue and ensure safety	[33,52,53,54,55,56,57]
Comparison with Traditional Power Training Methods	Olympic weightlifting emphasizes maximal power and strength with longer recovery, making it ideal for lower-body power development. With plyometric integration, HIIT is more accessible and enhances anaerobic capacity and neuromuscular efficiency. Strategic off-season focus on Olympic lifts followed by in-season HIIT improves power endurance.	Olympic weightlifting, Plyometric HIIT	Optimizes power endurance and sport-specific explosiveness	Higher technical demands and longer recovery in Olympic lifts	[26,58,59,60,61,62]
Integrated Approach for Power Development	Integrated HIIT and traditional power training enhance fast-twitch fiber activation, neuromuscular efficiency, and fatigue resistance. Effective integration through periodization prevents fatigue accumulation and interference effects, optimizing force production and power output.	Integrated HIIT with traditional power training, Periodized training cycles	Maximizes force production, sustained power output, and overall performance	Potential interference effects, if not periodized effectively	[26,59,60,61,62]

Abbreviations: RFD = rate of force development; SSC = stretch-shortening cycle; HIIT = high-intensity interval training.

**Table 4:** HIIT protocols for strength and power gains.

HIIT Protocol	Key Benefits	Effective Protocols	Challenges	References
Sprint Interval Training (SIT)	Enhances power, speed, and anaerobic capacity; improves linear speed, change in direction ability, and peak power output.	6–10 s of maximal sprints with 30–60 s rest. Progressive sprint-release model.	Limited endurance improvements for severe-intensity exercise. Requires optimized work-to-rest ratios.	[63,64,65,66,67]
Resistance-Based HIIT	Improves lower and upper body explosive strength; enhances neuromuscular efficiency and power through weighted explosive movements.	High-Intensity Power Training (HIPT), Olympic lifting, Contrast training, Kettlebell exercises.	Limited by the absence of heavy progressive overload. Potential interference with traditional strength gains.	[38,68,69,70,71,72]
Plyometric and Agility-Integrated HIIT	Boosts elastic strength, reactive power, and rapid force production; improves sprint speed, change-of-direction speed, and injury prevention.	Combined with sprint exercises; Vertical, lateral, and horizontal jumps. Long-term high-intensity plyometrics.	It requires proper monitoring and individualization to prevent injury. There is a high demand for neuromuscular systems.	[45,52,55,57,73,74]
Sport-Specific HIIT Applications	Enhances speed, aerobic, and anaerobic capacity; improves power and sport-specific strength for various sports.	Sled pushes, grappling drills, short-duration high-intensity efforts, multi-directional sprints.	Risk of excessive fatigue. Requires sport-specific adaptation and adequate recovery.	[21,26]

Abbreviations: HIPT = high-intensity power training; SIT = sprint interval training; HIIT = high-intensity interval training.

**Table 5:** Key focus on HIIT practical considerations and periodization strategies.

Key Focus	Key Insights	References
Balancing HIIT with Strength Training	Integrating HIIT with strength training requires managing volume, intensity, and timing to prevent interference. Short-duration, high-intensity efforts combined with blood-flow restriction can enhance anaerobic adaptations and sprint performance without significantly impacting aerobic capacity. Strategic scheduling of HIIT sessions relative to strength training optimizes force production and minimizes fatigue-related impairments.	[16,75,76]
Fatigue Management and Recovery Strategies	HIIT induces significant neuromuscular fatigue, necessitating effective recovery strategies. Utilizing Heart Rate Variability (HRV) and Rate of Perceived Exertion (RPE) provides insights into recovery status. Active recovery methods, nutritional support, and sleep optimization are critical for managing central and peripheral fatigue, ensuring sustained performance gains.	[21,25,77,78,79,80]
Individualization of HIIT Protocols	Tailoring HIIT protocols based on athlete-specific needs, such as training experience and sports demands, maximizes neuromuscular adaptations. Lower-intensity HIIT with extended recovery benefits beginners, while high-intensity protocols with shorter recovery suit elite athletes. Individualizing HIIT using metrics like anaerobic speed reserve ensures uniform adaptations across diverse athletic profiles.	[45,81,82,83,84,85,86]
Periodization Strategies for HIIT	Implementing periodized HIIT protocols, adjusting intensity, volume, and recovery optimizes neuromuscular adaptations while minimizing fatigue and injury risks. Effective periodization ensures that HIIT complements traditional strength training, facilitating long-term power, endurance, and overall performance improvements.	[26,86]

Abbreviations: HRV = heart rate variability; RPE = rate of perceived exertion; HIIT = high-intensity interval training.

## Data Availability

Not applicable.
